# Propensity score-matching analysis comparing laparoscopic and open pancreaticoduodenectomy in elderly patients

**DOI:** 10.1038/s41598-019-49455-9

**Published:** 2019-09-10

**Authors:** Heeji Shin, Ki Byung Song, Young Il Kim, Young-Joo Lee, Dae Wook Hwang, Jae Hoon Lee, Sang Hyun Shin, Jaewoo Kwon, Shadi Alshammary, Guisuk Park, Yejong Park, Seung Jae Lee, Song Cheol Kim

**Affiliations:** Division of Hepatobiliary and Pancreatic Surgery, Department of Surgery, University of Ulsan College of Medicine and Asan Medical Center, Seoul, South Korea

**Keywords:** Pancreatic cancer, Pancreatic cancer

## Abstract

There is little evidence on the safety and benefits of laparoscopic pancreaticoduodenectomy (LPD) in elderly patients; therefore, we evaluated the feasibility and efficacy of this procedure by comparing perioperative and oncological outcomes between LPD and open pancreaticoduodenectomy (OPD) in elderly patients. We retrospectively reviewed the data of 1,693 patients who underwent PD to manage periampullary tumours at a single institution between January 2014 and June 2017. Of these patients, 326 were elderly patients aged ≥70 years, with 56 patients allocated to the LPD group and 270 to the OPD group. One-to-one propensity score matching (56:56) was used to match the baseline characteristics of patients who underwent LPD and OPD. LPD was associated with significantly fewer clinically significant postoperative pancreatic fistulas (7.1% vs. 21.4%), fewer analgesic injections (10 vs. 15.6 times; p = 0.022), and longer operative time (321.8 vs. 268.5 minutes; p = 0.001) than OPD in elderly patients. There were no significant differences in 3-year overall and disease-free survival rates between the LPD and OPD groups. LPD had acceptable perioperative and oncological outcomes compared with OPD in elderly patients. LPD is a reliable treatment option for elderly patients with periampullary tumours.

## Introduction

In 2015, the average life expectancy at birth of the global population was 71 years, according to Global Health Observatory data released by the World Health Organization. The Korea National Statistical Office recently released a report that senior citizens aged 70 years and over currently constitute 14.1% of the Korean population, and this percentage is expected to reach 20% by 2026^1^. Similar growth of the aging population is expected to become an important issue worldwide.

It has been well known that the risk of developing periampullary cancer increases with age^2–4^. With the increase in life expectancy, the number of elderly patients requiring surgery will also increase, and the number of patients aged 70 years and older with resectable periampullary tumours is predicted to increase in the future.

Pancreaticoduodenectomy (PD) is one of the most challenging surgeries because it involves complex anatomy and necessitates numerous reconstructions. Furthermore, although laparoscopic surgery reduces surgery-related morbidity after various procedures^5,6^, laparoscopic pancreaticoduodenectomy (LPD) is a complex procedure, and it is unclear whether it confers any benefits. The general consensus is that LPD tends to yield better outcomes than open pancreaticoduodenectomy (OPD) when performed at a high-volume centre^7–10^. However, the safety of LPD has been widely criticised, as the majority of hospitals performing the procedure are low-volume centres, where it has been associated with increased morbidity and mortality^11–13^. Despite this controversy, the studies comparing LPD and OPD have been gradually increased.

As moving towards a rapidly ageing society, laparoscopic surgery as treatment for colorectal and gastric cancer has been reported its effectiveness and safety in elderly patients^14–16^, but there is little evidence on the safety and benefits of LPD in elderly patients with periampullary tumours. The present study aimed to compare perioperative and oncological outcomes between LPD and OPD in elderly patients using propensity score-matching analysis.

## Results

We performed 1,693 consecutive PDs (262 LPDs, 39 robotic PDs, 1,392 OPDs) in patients with periampullary tumours at our institution between January 2014 and June 2017. Of these patients, 326 (146 women, 180 men) were elderly patients who were undergoing PD for resectable periampullary tumours, with a mean age of 74.6 years (standard deviation [SD]: 3.5 years), a mean body mass index (BMI) of 23.5 kg/m^2^ (SD: 8.4 kg/m^2^), and mean American Society of Anesthesiologists (ASA) score of 2.1 (SD: 0.4). Histological diagnosis was confirmed in all patients, and the most common indication for PD was distal common bile duct cancer (n = 111; 34%), followed by pancreatic cancer (n = 94; 28.8%) and ampulla of Vater cancer (n = 67; 20.6%). The mean operative time was 294.3 minutes (SD: 67.9 minutes), and the mean length of postoperative hospital stay (POHS) was 16 days (SD: 13.1). The overall complication rate was 57.7%. Most patients had either no postoperative complications (n = 138; 42.3%) or minor Clavien–Dindo grade I/II events (n = 152; 46.6%). Postoperative pancreatic fistula (POPF) was the most frequent complication, with 20.2% of patients (n = 66) having clinically significant (grade B/C) pancreatic fistula. Major morbidity (Clavien–Dindo grades III/IV/V) occurred in 36 patients (11%). No 90-day mortality occurred.

### Overall comparison between LPD and OPD in terms of perioperative outcomes in elderly patients

Of the 326 elderly patients who underwent PD, 56 (17.2%) underwent LPD and 270 (82.8%) underwent OPD. The demographic data and perioperative outcomes of patients in both groups are presented in Table 1. Patients in the LPD group had lower BMI (22.8 vs. 23.6 kg/m^2^; p = 0.045), lower rate of preoperative biliary drainage (55.4% vs. 73.3%; p = 0.07), and a lower incidence of pancreatic cancer (25% vs. 42.6%; p = 0.0174). The LPD group had longer operative time than the OPD group (321.8 vs. 288.6 minutes; p < 0.001). There were no significant differences in age, sex, ASA score, or POHS between the two groups.

**Table 1:** Demographic data and perioperative and pathological outcomes in 326 elderly patients who underwent LPD and OPD.

Variables	LPD (n = 56)	OPD (n = 270)	p-value
Age (years) ± SD	74.8 ± 3.7	74.6 ± 3.5	0.703
Sex (male:female)	27:29	153:117	0.248
Body mass index (kg/m^2^) ± SD	22.8 ± 2.6	23.6 ± 2.9	**0.045**
American Society of Anesthesiologists score ± SD	2.1 ± 0.5	2.1 ± 0.4	0.987
Preoperative biliary drainage, n (%)	31 (55.4)	198 (73.3)	**0.007**
Operative time (min) ± SD	321.8 ± 56.1	288.6 ± 68.8	**<0.001**
Pathology, n (%)			**0.017**
Pancreatic cancer	14 (25%)	115 (42.6%)	
Distal common bile duct cancer	19 (33.9%)	92 (34.1%)	
Ampulla of Vater and duodenal cancer	23 (41.1%)	63 (23.3%)	
Postoperative hospital stay (days) ± SD	13.5 ± 11.3	16.5 ± 11.3	0.12

Abbreviations: LPD, laparoscopic pancreaticoduodenectomy; OPD, open pancreaticoduodenectomy; SD, standard deviation.

Table 2 presents the in-hospital complications in both groups, classified and graded according to the Clavien–Dindo system. The LPD group showed a significant lower overall complication rate (35.8% vs. 62.2%; p < 0.001). However, the rate of major complications (Clavien–Dindo grade III–V) did not differ between the two groups (5.4% in the LPD group vs. 12.2% in the OPD group; p = 0.136). The most common postoperative complication was POPF in both groups, with a higher incidence in the OPD group (p < 0.001). Finally, the rate of clinically significant pancreatic fistula (grade B/C) was significantly lower in the LPD group (7.1%) than in the OPD group (23%; p = 0.007).

**Table 2:** In-hospital complications in 326 elderly patients who underwent LPD and OPD.

Surgical complication according to Clavien–Dindo classification	LPD (n = 56)	OPD (n = 270)	
No complication	36	102	
Grade I	10	63	
Chylous ascites treated with low-long-chain triglyceride diet	9	56	
Superficial wound infection treated with bedside care	1	7	
Grade II	7	72	
Antibiotic therapy for intra-abdominal fluid collection	4	46	
Ileus		9	
Delayed gastric emptying		3	
Pneumonia		3	
Atrial fibrillation		4	
Pseudomembranous colitis		3	
Postoperative pancreatitis		1	
Uncontrolled ascites in patients with liver cirrhosis	1	1	
Bile leakage treated with conservative management		1	
Postoperative delirium		1	
Grade III	0	24	
Grade IIIa		19	
Grade B pancreatic fistula with drainage		14	
Pseudoaneurysmal bleeding treated with embolization		1	
Bile leakage treated with interventional therapy		2	
Pulmonary artery thromboembolism (inferior vena cava			
filter insertion)		1	
Delayed gastric emptying (duodenojejunostomy site stenosis treated with balloon dilatation)		1	
Grade IIIb		5	
Wound dehiscence		4	
Mechanical ileus with adhesiolysis		1	
Grade IV (intensive care unit treatment)	3	6	
Grade C pancreatic fistula (pancreaticojejunostomy revision)	1	1	
Intra-abdominal fluid collection with sepsis	1		
Pseudoaneurysmal bleeding (stent graft insertion)		1	
Pseudoaneurysmal bleeding (embolization)	1		
Pneumonia with respiratory failure		2	
Air embolism and heart failure		1	
Atrial fibrillation		1	
Grade V	0	3	
Aspiration pneumonia		1	
Septic shock with gastric perforation due to ischemia		1	
Postoperative bleeding with duodenojejunostomy disruption		1	
Surgical complication according to Clavien–Dindo classification	20 (35.7%)	168 (62.2%)	**<0.001**
Grade I	10	63	
Grade II	7	72	
Grade III	0	24	
Grade IV	3	6	
Grade V	0	3	
Major morbidity (≥Grade III)	3 (5.4%)	33 (12.2%)	0.136
Pancreatic fistula by ISGPS			
Biochemical leakage	12 (21.4%)	87 (32.2%)	
Grade B	3 (5.6%)	61 (22.6%)	
Grade C	1 (1.8%)	1 (0.4%)	
Overall pancreatic fistula	16 (28.6%)	149 (55.2%)	**<0.001**
Clinically significant pancreatic fistula	4 (7.1%)	62 (23%)	**0.007**

Abbreviations: LPD, laparoscopic pancreaticoduodenectomy; OPD, open pancreaticoduodenectomy; ISGPS, International Study Group of Pancreatic Surgery.

### Propensity score-matched comparison of perioperative outcomes in elderly patients who underwent PD (LPD vs. OPD)

To control for selection bias, one-to-one propensity score matching was used (56 patients in each group), adjusted for sex, age, BMI, ASA score, preoperative biliary drainage, and pathologies, so that all baseline parameters were balanced after matching, as shown in Table 3.

**Table 3:** Baseline parameters in the propensity score-matched groups.

Variables	LPD (n = 56)	OPD (n = 56)	p-value
Age (years) ± SD	74.8 ± 3.7	74.7 ± 3.5	0.99
Sex (male:female)	27:29	25:31	0.71
Body mass index (kg/m^2^) ± SD	22.8 ± 2.6	22.6 ± 2.3	0.75
ASA score (mean) ± SD	2.1 ± 0.5	2.1 ± 0.4	1
Preoperative biliary drainage, n (%)	31 (55.4)	33 (58.9)	0.85
Pathology, n (%)			1
Pancreatic cancer	14 (25)	14 (25)	
Ampulla of Vater or duodenal cancer	23 (41.1)	23 (41.1)	
Distal common bile duct cancer	19 (33.9)	19 (33.9)	

Abbreviations: LPD, laparoscopic pancreaticoduodenectomy; OPD, open pancreaticoduodenectomy; BMI, body mass index; ASA, American Society of Anesthesiologists; SD, standard deviation.

The perioperative outcomes are summarised in Table 4. The LPD group had a significantly longer mean operative time than the OPD group (321.8 vs. 268.5 minutes; p < 0.001). The mean number of analgesic injections was lower in the LPD group than in the OPD group (9.6 vs. 14.3; p < 0.026). There were no significant differences in estimated blood loss, soft diet starting time, and POHS.

**Table 4:** Operative and perioperative outcomes.

	LPD (n = 56)	OPD (n = 56)	p-value
Operative time (min) ± SD	321.8 ± 56.1	268.5 ± 70.5	**<0.001**
Estimated blood loss (mL) ± SD	468 ± 331	362 ± 363	0.11
Soft diet starting time (postoperative day) ± SD	5.6 ± 3	5.7 ± 6.3	0.94
Postoperative hospital stay (day) ± SD	13.5 ± 11.3	15.7 ± 12.7	0.323
Number of analgesic injections, n ± SD	9.6 ± 8.5	14.3 ± 13.1	**0.026**
Surgical complication according to Clavien–Dindo classification	20 (35.7%)	32 (57.1%)	**0.023**
Grade I	10	12	
Grade II	7	14	
Grade III	0	4	
Grade IV	3	2	
Major morbidity	3 (5.4%)	6 (10.7%)	0.297
Readmission	9	5	0.253
Pancreatic fistula by ISGPS			
Biochemical leakage	12 (21.4%)	23 (41.1%)	
Grade B	3 (5.4%)	12 (21.4%)	
Grade C	1 (1.8%)	0	
Overall pancreatic fistula	16 (28.6%)	35 (62.5%)	**<0.001**
Clinically significant pancreatic fistula	4 (7.1%)	12 (21.4%)	**0.031**

Clavien–Dindo grade > III was defined as a major complication.

Pancreatic fistula was graded according to the modified 2016 ISGPS.

Clinical effect of biochemical leakage grades B/C indicated clinically significant pancreatic fistula.

Abbreviations: LPD, laparoscopic pancreaticoduodenectomy; OPD, open pancreaticoduodenectomy; ISGPS, International Study Group of Pancreatic Surgery; SD, standard deviation.

Table 4 also presents the postoperative complications classified and graded according to the Clavien–Dindo classification system in the two groups. The overall morbidity rate was significantly lower in the LPD group than in the OPD group (35.7% vs. 57.1%, p = 0.023). There was no significant difference in major complication (Clavien–Dindo grade III–V) rate between the two groups (5.4% vs. 10.7%; p = 0.297).

The most common postoperative complication was POPF in both groups, with a higher incidence in the OPD group (p < 0.001). Four of 56 patients (7.1%) in the LPD group and 12 of 56 patients (21.4%) in the OPD group developed a clinically significant pancreatic fistula (p = 0.031).

### Comparison of oncological outcomes and survival in elderly patients with periampullary tumours in the LPD and OPD groups

**Figure 1:**
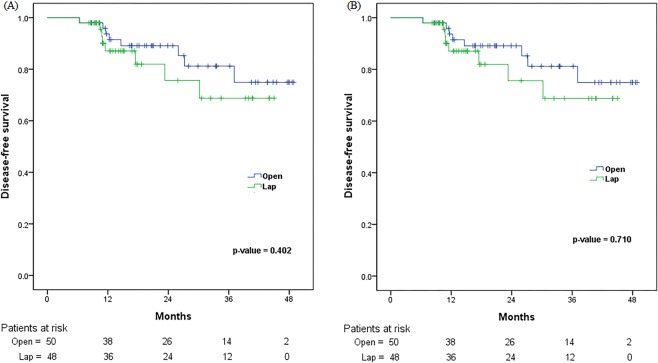
Comparison of Kaplan–Meier survival curve for (**A**) overall survival and (**B**) disease-free survival between open and laparoscopic pancreaticoduodenectomy.

Table 5 shows the oncological outcomes in elderly patients with periampullary tumours. There was no difference in microscopic positive margin (R1) rate between the two groups (LPD: 10.2% vs. OPD: 6%; p = 0.443). In the LPD group, five patients had positive resection margin (RM) findings. Three had an RM-positive hepatic duct (two with high-grade dysplasia, one with adenocarcinoma). Another two patients had periductal radial RM-positive findings. In the OPD group, three patients had RM-positive findings. Two had an RM-positive hepatic duct (one with high-grade dysplasia, one with adenocarcinoma). Another one patient had pancreatic radial RM-positive findings. The numbers of lymph nodes in the resected specimens did not differ between the two groups (LPD: 15.3 vs. OPD: 17.7; p = 0.152). The 3-year overall survival rates of patients in the LPD and OPD groups were 68.8% and 83.2%, respectively (p = 0.383). The 3-year disease-free survival rates of patients in the LPD and OPD groups were 53.3% and 65.6%, respectively (p = 0.71). There were no differences in Kaplan–Meier curves of overall and disease-free survival between the two groups (Fig. 1).

**Table 5:** Comparison of oncological outcomes in elderly patients with periampullary tumours in the LPD and OPD groups.

	LPD (n = 49)	OPD (n = 50)	p-value
Diagnosis			
Pancreatic cancer	8	10	
Ampulla of Vater cancer	18	18	
Distal common bile duct cancer	19	19	
Duodenal cancer	4	3	
Age (years) ± SD	74.9 ± 3.7	74.6 ± 3.5	0.703
Sex (male:female)	26:23	23:27	0.482
Tumour size (cm) ± SD	2.7 ± 1.2	2.6 ± 1.2	0.623
Rate of positive LN, n (%)	17 (34.7%)	21 (42%)	0.455
Total number of LN, n ± SD	15.3 ± 8.7	17.7 ± 8.3	0.152
Surgical margin, n (%)			0.443
R0	44 (89.8)	47 (94)	
R1	5 (10.2)	3 (6)	
Perineural invasion, n (%)			0.257
Yes	18 (36.7)	24 (48)	
No	31 (62.3)	26 (52)	
Lymphovascular invasion, n (%)			0.916
Yes	23 (46.9)	24 (48)	
No	26 (53.1)	26 (52)	
Differentiation, n (%)			0.413
Well differentiated	9 (18.4)	11 (22)	
Moderately differentiated	29 (59.2)	27 (54)	
Poorly differentiated	9 (18.4)	6 (12)	
Unknown	2 (4)	6 (12)	
3-year overall survival rate, (%)	68.8	83.2	0.383
3-year disease-free survival rate, (%)	53.3	65.6	0.71

Abbreviations: LPD, laparoscopic pancreaticoduodenectomy; OPD, open pancreaticoduodenectomy; SD, standard deviation; LN, lymph nodes; R0, negative resection margin; R1, positive resection margin.

## Discussion

Populations are aging worldwide, with individuals living longer and the surgical needs of elderly patients increasing. In particular, elderly patients show higher prevalence of multiple morbidities and geriatric syndromes and lower physiological reserve and preoperative nutritional conditioning than those of young patients^17,18^. Several reports have found that age itself is an important risk factor for postoperative morbidity and mortality, and that it decreases the tolerability for surgical stress^19–21^. Minimally invasive surgery has proven to be safe and effective and has largely replaced open surgery in many procedures^8,22–24^. The typical benefits of laparoscopic surgery include reduced postoperative pain and POHS length, improved mobilization, faster return to normal activity, and fewer abdominal wall complications^22,25–27^. Because of these benefits of laparoscopic surgery, LPD can be considered as alternative surgical option for elderly patients. However, it is questionable whether the advantages of laparoscopic surgery are applicable to LPD performed in elderly patients.

In many large volume hospitals, improved outcomes after LPD have broadened the selection criteria for surgery, leading to the inclusion of more elderly patients, and the procedure is becoming increasingly popular among experienced pancreatic surgeons^8^. Thus, researchers must establish whether LPD is a suitable surgery in elderly patients by researching this matter in large-volume hospitals.

To evaluate the feasibility and efficacy of LPD in elderly patients with periampullary tumours, we compared perioperative and oncological outcomes between LPD and OPD in elderly patients aged ≥70 years. To our knowledge, this was the first large-scale cohort study including elderly patients that investigated the clinical feasibility of LPD to manage periampullary tumours. Although a randomised controlled trial (RCT) would be ideal to address this matter, the rarity of periampullary tumours as a surgical indication in elderly patients may prompt investigators to avoid including frail patients in prospective randomised trials. To circumvent this impracticability, we conducted a propensity score-matched study.

PD is one of the most complex general surgical procedures involving various anastomoses, and POPF caused by failure of pancreatic enteric anastomosis is the most common complication of PD^28,29^. In the present study, the overall and clinically significant rates of POPF in the LPD group were lower than those in the OPD group (p < 0.001 and p < 0.031). Previous studies have reported that laparoscopic surgery leads to similar or superior perioperative outcomes with regards to POPF^8,10,30–32^. Most surgeons recognise from experience that the development of bowel wall oedema plays a key role in anastomotic failure. One comparative study evaluated the development of bowel wall oedema during laparoscopic and open visceral surgeries, showing that laparoscopic surgery is associated with lower rates of this finding^33^. The authors concluded that prevention of bowel wall oedema may be one advantage of minimally invasive surgery, as it leads to faster anastomotic healing. Nonetheless, since there was no evidence-based data, we need further research of the effects of laparoscopic surgery on anastomotic healing in the future.

Postoperative ileus and delayed gastric emptying (DGE) are the most common complications of PD. Laparoscopic surgery is associated with a lower rate of postoperative ileus than open surgery^34^, and several studies have reported that clinically significant DGE was less frequent in LPD than in OPD^12^. These decreased complication rates may occur because LPD involves minimal manipulation of the bowel and is associated with less postoperative adhesion.

In any case, it is still unclear whether laparoscopic surgery affects the incidence of postoperative pneumonia. In several studies, fewer postoperative pulmonary complications developed after laparoscopic surgery than after open surgery^35–37^. In this regard, diaphragmatic splinting due to postoperative pain may lead to the increased incidence of pneumonia after open surgery^38^. In a previous study, we reported the obvious benefits of LPD, including significantly less postoperative pain and faster recovery^8^. Indeed, in the present study, the mean number of analgesic injections was lower in the LPD group than in the OPD group (9.6 vs. 14.3; p < 0.026). Ambulation with an erect posture tends to exacerbate abdominal pain, especially after OPD involving a larger incision. In elderly patients, early ambulation after surgery should be encouraged, and delays in this regard have been related to the development of pneumonia and to increased POHS length^39,40^. In patients who have undergone OPD, severe abdominal pain likely causes such a delay, leading to the high rate of complications in such patients, including pneumonia. However, our data were inadequate to show any statistically significant benefits of LPD to in the prevention of postoperative pulmonary complications.

Moreover, we evaluated our results to determine whether the survival data justify the implementation of LPD in elderly patients. In particular, the important operative oncological measures of PD include the retroperitoneal margin, superior mesenteric artery margin, number of resected lymph nodes, and survival^41,42^. The number of harvested lymph nodes and the microscopic negative margin (R0) resection rate were not different between the LPD and OPD groups. The mean number of lymph nodes in the resected specimens was 15.3 in the LPD group and 17.7 in the OPD group, which is consistent with the consensus adequate lymph node retrieval number^43^. In addition, median overall survival and relapse-free survival were not significantly different between the LPD and OPD groups. The laparoscopic approach was similar to the open procedure with respect to oncological surrogates and survival in elderly patients with periampullary tumours.

In conclusion, the results of the present study indicated that LPD in elderly patients leads to acceptable postoperative and oncological outcomes, justifying the use of LPD in elderly patients with periampullary tumours.

## Methods

The present study was a single-centre, retrospective, propensity score-matched case–control study that included patients who underwent PD for periampullary tumours between January 2014 and June 2017 at Asan Medical Center, Seoul, Korea. The study was approved by the institutional review board of Asan Medical Center (Approval number: 2018‐0486). It was exempted from the need for informed consent for the following reasons: (1) the risk expected was not greater than Level I; (2) Gaining consent from the research participants was deemed practically impossible during the course of the research, and it may have seriously affected the validity of the research; (3) There was no reason to assume the subjects would refuse consent, and the risks to the subjects were extremely low, even after the need for consent was waived; (4) The exemption from consent did not infringe upon the rights or welfare of the subject; (5) The study was not carried out to gain approval of medicines or medical devices, and it was not regulated by regulatory agencies. All experiments were performed in accordance with relevant guidelines and regulations.

Data were collected on patient demographics, preoperative management, operative variables, postoperative outcomes, pathological findings, and postoperative follow-up details, including survival status. The collected demographic data included age, sex, BMI in kg/m^2^, and ASA score. Operative details obtained from the anaesthesia record included surgical approach (laparoscopic/open), operative time (from incision to wound closure), estimated blood loss, and packed red blood cell transfusion. Estimated blood loss was calculated using the equation from a previous paper^44^.

The collected pathological specimen data included final pathological diagnosis, tumour size at its largest diameter, and margin status. The outcomes used to assess the oncological adequacy of LPD included oncological surrogates, such as pathological measures (i.e., number of harvested lymph nodes) and R0 vs. R1. RMs were classified by a pathologist, according to previous reports, as follows: R0, no cancer cells or high-grade dysplasia seen microscopically at the resection margin; R1, cancer cells or high-grade dysplasia present microscopically at the resection margin or within 1 mm of the margin^45^. For staging, we referred to the TNM Classification of Malignant Tumours, 7^th^ edition, published by the Union for International Cancer Control.

**Figure 2:**
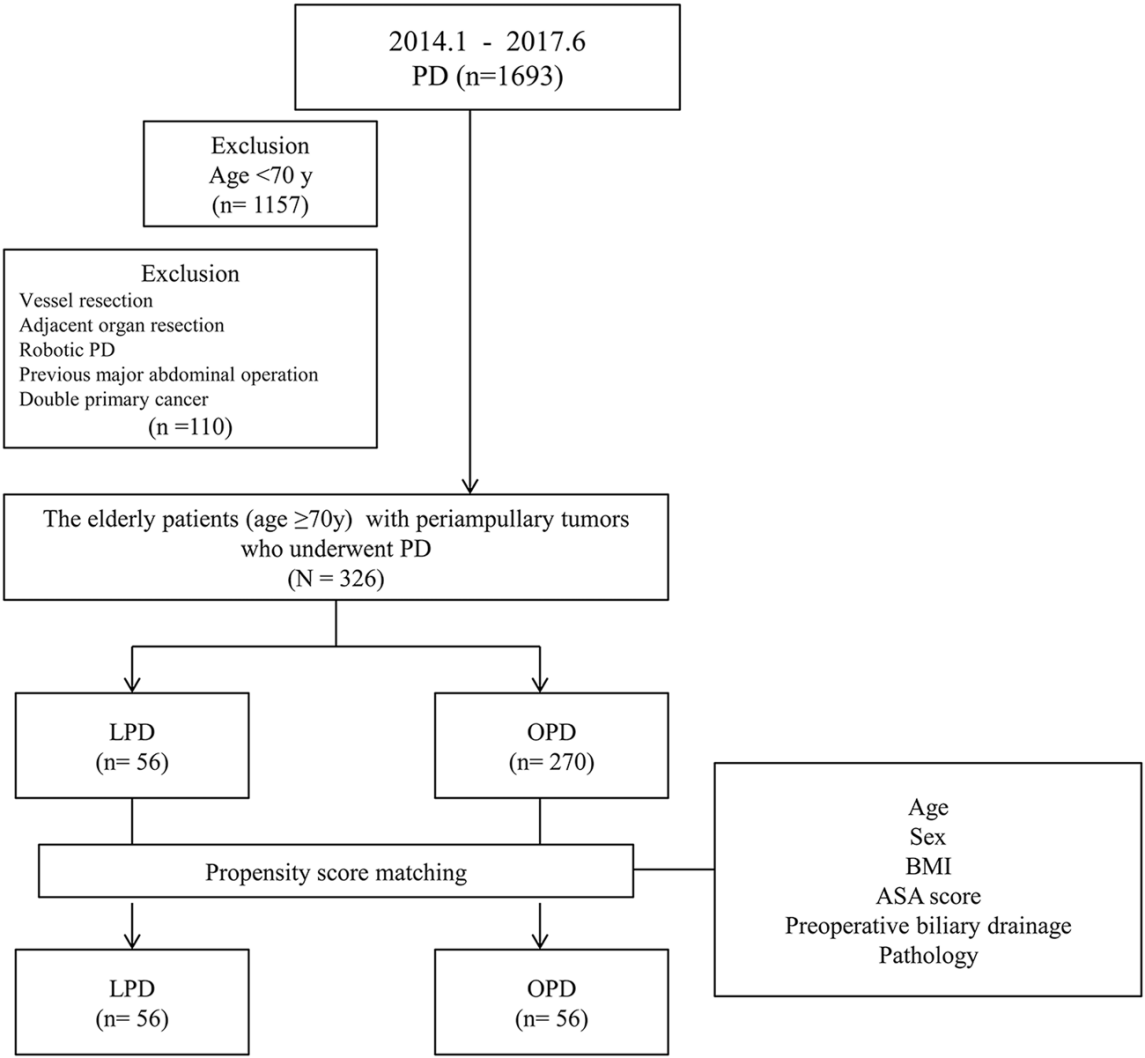
Flow chart of patient inclusion. The study enrolled 326 patients aged ≥70 years who underwent PD to manage periampullary tumours. The patients were divided into two operative groups for analysis: 56 patients in the LPD group and 270 in the OPD group. To control for selection bias, a comparative study was performed in 112 patients using one-to-one propensity-score matching (56 patients in the LPD group, 56 in the OPD group). Abbreviations: PD, pancreaticoduodenectomy; LPD, laparoscopic pancreaticoduodenectomy; OPD, open pancreaticoduodenectomy; BMI, body mass index; ASA, American Society of Anesthesiologists.

Our hospital is a high-volume centre, and 1,693 consecutive PDs were performed in patients with periampullary tumours by experienced surgeons between January 2014 and June 2017. We defined the patient over the age of 70 as elderly patient. Ultimately, the study included 326 patients over 70 years of age who had resectable periampullary tumours who underwent curative PD. We excluded 1,257 patients for the following reasons: age <70 years, major vessel invasion, adjacent organ invasion, robotic PD, double primary cancer, and previous major abdominal surgery (Fig. 2). The remaining elderly patients who underwent PD to manage periampullary tumours at our institution were divided into the following two operative groups for analysis: 56 patients in the LPD group and 270 patients in the OPD group. To control for selection bias, a comparative study was performed in 112 patients using one-to-one propensity score matching (56 patients in the LPD group, 56 in the OPD group). The primary end point of the study was perioperative outcomes.

Postoperative complications were graded according to the Clavien–Dindo classification system (minor complications: grades I and II, major complications: grades III–V)^46^. The highest complication grade in each patient was considered the final overall complication grade. POPFs were defined according to the modified 2016 International Study Group of Pancreatic Surgery consensus definitions^47^. DGE was defined as the inability to tolerate oral intake, emesis, and the need for prokinetics or nasogastric tube decompression, with grades A, B, and C based on the presence and duration of each of these factors^48^. In the present study, the patients chose which surgical approach they preferred after surgeons had adequately explained the risks and benefits of each.

### Surgical technique

#### LPD

The surgical technique of LPD has been described in a previous paper^2^.

#### OPD

The patient was placed in the supine position. The surgery usually required a long midline or inverted L incision. All reconstructions were performed in the same way as for LPD.

### Statistical analysis

Propensity scores were estimated using logistic regression. The following covariates were included in the regression model^49,50^: age, sex, BMI, ASA score, preoperative biliary drainage, and pathologies (tumours of the pancreas, ampulla of Vater, and distal common bile duct). These covariates were selected because they can affect the choice of surgical approach and perioperative outcomes. The operative approach (laparoscopic vs. open) was entered into the regression model as a dependent variable. A 1:1 “nearest neighbour”, case–control match without replacement was used^51,52^. Each patient treated using laparoscopic surgery was matched with the patient treated using open surgery who had the closest estimated propensity scores. Post-match baseline characteristics and operative and postoperative variables were compared between the groups using bivariate analysis. Categorical data were reported as either number or percentage and significance tested using two-tailed Fisher’s exact test or the chi-square test. Continuous variables were expressed as mean and standard deviation (SD). A p-value of <0.05 was used to determine significance.

Overall survival was defined as the time interval between the date of surgery and the date of death and was censored at the last follow-up date for patients who were alive. All patients were accounted for in the follow-up. Disease-free survival was defined as the time interval between the date of surgery and the date of recurrence or death, whichever came first, and was censored at the last follow-up date for patients who were alive without recurrence. Statistical analyses were performed using SPSS version 21.0 (IBM Corp., Armonk, NY, USA).

## References

[1] Hyun KR, Kang S, Lee S (2016). Population Aging and Healthcare Expenditure in Korea. Health economics.

[2] Rawla P, Sunkara T, Gaduputi V (2019). Epidemiology of Pancreatic Cancer: Global Trends, Etiology and Risk Factors. World journal of oncology.

[3] Jackson, S. S. *et al*. Anthropometric Risk Factors for Cancers of the Biliary Tract in the Biliary Tract Cancers Pooling Project. *Cancer research* (2019). 10.1158/0008-5472.CAN-19-0459 PMC6759233 31113819

[4] Ramai D (2019). Demographics, tumor characteristics, treatment, and clinical outcomes of patients with ampullary cancer: a Surveillance, Epidemiology, and End Results (SEER) cohort study. Minerva gastroenterologica e dietologica.

[5] Hida K (2018). Open versus Laparoscopic Surgery for Advanced Low Rectal Cancer: A Large, Multicenter, Propensity Score Matched Cohort Study in Japan. Ann Surg.

[6] Park YK (2018). Laparoscopy-assisted versus Open D2 Distal Gastrectomy for Advanced Gastric Cancer: Results From a Randomized Phase II Multicenter Clinical Trial (COACT 1001). Ann Surg.

[7] Conrad C (2017). Comparable long-term oncologic outcomes of laparoscopic versus open pancreaticoduodenectomy for adenocarcinoma: a propensity score weighting analysis. Surg Endosc.

[8] Song KB (2015). Matched case-control analysis comparing laparoscopic and open pylorus-preserving pancreaticoduodenectomy in patients with periampullary tumors. Ann Surg.

[9] Palanivelu C (2017). Randomized clinical trial of laparoscopic versus open pancreatoduodenectomy for periampullary tumours. Br J Surg.

[10] Asbun HJ, Stauffer JA (2012). Laparoscopic vs open pancreaticoduodenectomy: overall outcomes and severity of complications using the Accordion Severity Grading System. J Am Coll Surg.

[11] Adam MA (2015). Minimally Invasive Versus Open Pancreaticoduodenectomy for Cancer: Practice Patterns and Short-term Outcomes Among 7061 Patients. Ann Surg.

[12] Zhao Z (2017). A systemic review and an updated meta-analysis: minimally invasive vs open pancreaticoduodenectomy. Sci Rep.

[13] van Hilst J (2019). Laparoscopic versus open pancreatoduodenectomy for pancreatic or periampullary tumours (LEOPARD-2): a multicentre, patient-blinded, randomised controlled phase 2/3 trial. Lancet Gastroenterol Hepatol.

[14] Roscio F (2016). Is laparoscopic surgery really effective for the treatment of colon and rectal cancer in very elderly over 80 years old? A prospective multicentric case-control assessment. Surg Endosc.

[15] Zong L (2017). Feasibility of laparoscopic gastrectomy for elderly gastric cancer patients: meta-analysis of non-randomized controlled studies. Oncotarget.

[16] Antoniou SA, Antoniou GA, Koch OO, Pointner R, Granderath FA (2015). Laparoscopic colorectal surgery confers lower mortality in the elderly: a systematic review and meta-analysis of 66,483 patients. Surg Endosc.

[17] Chesney T, Acuna SA (2015). Do elderly patients have the most to gain from laparoscopic surgery?. Ann Med Surg (Lond).

[18] Alvis BD, Hughes CG (2015). Physiology Considerations in Geriatric Patients. Anesthesiol Clin.

[19] Eguchi T (2017). Impact of Increasing Age on Cause-Specific Mortality and Morbidity in Patients With Stage I Non-Small-Cell Lung Cancer: A Competing Risks Analysis. J Clin Oncol.

[20] Turrentine FE, Wang H, Simpson VB, Jones RS (2006). Surgical risk factors, morbidity, and mortality in elderly patients. J Am Coll Surg.

[21] Kim S, Brooks AK, Groban L (2015). Preoperative assessment of the older surgical patient: honing in on geriatric syndromes. Clin Interv Aging.

[22] Veldkamp R (2005). Laparoscopic surgery versus open surgery for colon cancer: short-term outcomes of a randomised trial. Lancet Oncol.

[23] Wang W (2014). Laparoscopic versus open total gastrectomy for gastric cancer: an updated meta-analysis. PLoS One.

[24] Shin SH (2016). Appraisal of laparoscopic distal pancreatectomy for left-sided pancreatic cancer: A large volume cohort study of 152 consecutive patients. PLoS One.

[25] Xie M (2015). Laparoscopic Colorectal Resection in Octogenarian Patients: Is it Safe? A Systematic Review and Meta-Analysis. Medicine (Baltimore).

[26] Lacy AM (2002). Laparoscopy-assisted colectomy versus open colectomy for treatment of non-metastatic colon cancer: a randomised trial. Lancet.

[27] Croome Kristopher P., Farnell Michael B., Que Florencia G., Reid-Lombardo KMarie, Truty Mark J., Nagorney David M., Kendrick Michael L. (2014). Total Laparoscopic Pancreaticoduodenectomy for Pancreatic Ductal Adenocarcinoma. Annals of Surgery.

[28] Tabatabei SA, Hashemi SM (2015). Pancreatic anastomosis leakage management following pancreaticoduodenectomy how could be manage the anastomosis leakage after pancreaticoduodenectomy?. J Res Med Sci.

[29] Machado NO (2012). Pancreatic fistula after pancreatectomy: definitions, risk factors, preventive measures, and management-review. Int J Surg Oncol.

[30] Braga Marco, Vignali Andrea, Gianotti Luca, Zuliani Walter, Radaelli Giovanni, Gruarin Paola, Dellabona Paolo, Di Carlo Valerio (2002). Laparoscopic Versus Open Colorectal Surgery. Annals of Surgery.

[31] Hu Y (2016). Morbidity and mortality of laparoscopic versus open d2 distal gastrectomy for advanced gastric cancer: A randomized controlled trial. J Clin Oncol.

[32] Bonjer HJ (2015). A randomized trial of laparoscopic versus open surgery for rectal cancer. N Engl J Med.

[33] Marjanovic G (2014). A prospective clinical study evaluating the development of bowel wall edema during laparoscopic and open visceral surgery. J Gastrointest Surg.

[34] Lubawski J, Saclarides T (2008). Postoperative ileus: strategies for reduction. Ther Clin Risk Manag.

[35] Bablekos GD, Roussou T, Rasmussen T, Vassiliou MP, Behrakis PK (2003). Postoperative changes on pulmonary function after laparoscopic and open cholecystectomy. Hepatogastroenterology.

[36] Cone MM (2012). Effect of surgical approach on 30-day mortality and morbidity after elective colectomy: a NSQIP study. J Gastrointest Surg.

[37] Fujita T, Sakurai K (1995). Multivariate analysis of risk factors for postoperative pneumonia. Am J Surg.

[38] Grailey K (2013). Laparoscopic versus open colorectal resection in the elderly population. Surg Endosc.

[39] Fisher SR, Kuo YF, Graham JE, Ottenbacher KJ, Ostir GV (2010). Early ambulation and length of stay in older adults hospitalized for acute illness. Arch Intern Med.

[40] Kamel HK, Iqbal MA, Mogallapu R, Maas D, Hoffmann RG (2003). Time to ambulation after hip fracture surgery: relation to hospitalization outcomes. J Gerontol A Biol Sci Med Sci.

[41] Liu DN (2017). Superior mesenteric artery margin in pancreaticoduodenectomy for pancreatic adenocarcinoma. Oncotarget.

[42] Gnerlich JL (2012). Microscopic margins and patterns of treatment failure in resected pancreatic adenocarcinoma. Arch Surg.

[43] Jeyarajah DR (2014). Lymph node retrieval in pancreaticoduodenectomy specimens: does educating the pathologist matter?. HPB (Oxford).

[44] McCullough TC, Roth JV, Ginsberg PC, Harkaway RC (2004). Estimated blood loss underestimates calculated blood loss during radical retropubic prostatectomy. Urol Int.

[45] Schlitter AM, Esposito I (2010). Definition of microscopic tumor clearance (r0) in pancreatic cancer resections. Cancers (Basel).

[46] Dindo D, Demartines N, Clavien PA (2004). Classification of surgical complications: a new proposal with evaluation in a cohort of 6336 patients and results of a survey. Ann Surg.

[47] Bassi C (2017). The 2016 update of the International Study Group (ISGPS) definition and grading of postoperative pancreatic fistula: 11 Years After. Surgery.

[48] Wente MN (2007). Delayed gastric emptying (DGE) after pancreatic surgery: a suggested definition by the International Study Group of Pancreatic Surgery (ISGPS). Surgery.

[49] Austin PC (2011). An introduction to propensity score methods for reducing the effects of confounding in observational studies. Multivariate Behav Res.

[50] Heinze G, Juni P (2011). An overview of the objectives of and the approaches to propensity score analyses. Eur Heart J.

[51] Austin PC (2009). The relative ability of different propensity score methods to balance measured covariates between treated and untreated subjects in observational studies. Med Decis Making.

[52] Austin PC (2009). Balance diagnostics for comparing the distribution of baseline covariates between treatment groups in propensity-score matched samples. Stat Med.

